# Accuracy assessment of inverse distance weighting interpolation of groundwater nitrate concentrations in Bavaria (Germany)

**DOI:** 10.1007/s11356-022-22670-0

**Published:** 2022-09-03

**Authors:** Paul L. Ohlert, Martin Bach, Lutz Breuer

**Affiliations:** 1 Institute for Landscape Ecology and Resources Management (ILR), Research Centre for Biosystems, Land Use and Nutrition (iFZ), Justus Liebig University Giessen, Heinrich-Buff-Ring 26, 35392 Giessen, Germany; 2 Centre for International Development and Environmental Research (ZEU), Justus Liebig University Giessen, Senckenbergstrasse 3, 35390 Giessen, Germany

**Keywords:** Accuracy, Groundwater, Interpolation, Inverse distance weighting, Nitrate concentration, Random Forest model

## Abstract

For the designation of nitrate vulnerable zones under the EU Nitrate Directive, some German federal states use inverse distance weighting (IDW) as interpolation method. Our study quantifies the accuracy of IDW with respect to the designation of areas with a groundwater nitrate concentration above the threshold of 50 mg NO_3_/l using a dataset of 5790 groundwater monitoring sites in Bavaria. The results show that the absolute differences of nitrate concentrations between the monitoring sites are only weakly correlated within a range of no more than 0.4 km. The IDW cross-validated nitrate concentration of measurement sites shows a mean absolute error of 7.0 mg NO_3_/l and the number of measurement sites above 50 mg NO_3_/l is 44% too low by interpolation for all sites as a whole. The corresponding values for interpolation separately for the 18 hydrogeological regions in Bavaria are 7.1 mg NO_3_/l and 38%. The sensitivity and the accuracy of nitrate concentration maps due to the variation of IDW parameters and the position of sampling points are analysed by Monte Carlo IDW interpolations using a Random Forest modelled map as reference spatial distribution. Compared to this reference map, the area with a concentration above 50 mg NO_3_/l in groundwater is estimated by IDW to be 46% too low for the best IDW parametrization. Overall, IDW interpolation systematically underrates the occurrence of higher range nitrate concentrations. In view of these underestimations, IDW does not appear to be a suitable regionalization method for the designation of nitrate vulnerable zones, neither when applied for a federal state as a whole nor when interpolated separately for hydrogeological regions.

**Supplementary Information:**

The online version contains supplementary material available at 10.1007/s11356-022-22670-0.

## Introduction

Groundwater is the most important resource worldwide for supplying populations with drinking water (Foster and Chilton 2003). As in many other countries, the input of nitrate is one of the main causes of groundwater quality impairment in Germany (UBA 2017). In the European Union (EU), the Nitrate Directive 91/676/EEC and the Water Framework Directive (WFD) 2000/60/EC have been implemented to protect groundwater from nitrate pollution and to ensure the good status of groundwater bodies (GWB). The judgement of the European Court of Justice from June 21, 2018 declared that the German action programmes were inadequate and failed to fulfil the obligations of the Nitrate Directive. The consequence of that judgement was that the German Fertilizer Ordinance was revised in 2020. As a result, the German federal states are obliged to specify enhanced standards for regions with groundwater bodies that exceed the 50 mg NO_3_/l threshold or that show a trend of rising nitrate concentration as well as a concentration above 37.5 mg NO_3_/l. As a substantial requirement for those nitrogen-contaminated regions, nitrogen fertilization of field crops has to be reduced by 20% compared to the optimal fertilizer supply. This regulation has sparked massive protests from the agricultural sector. The designation of nitrate-contaminated regions (often referred to as ‘red areas’) can be performed with deterministic and geostatistic methods of regionalization (AVV GeA 2020). Inverse distance weighting (IDW) is used for this purpose by a number of German federal states.

The literature describes numerous methods for the transfer of point data to spatial data. Li and Heap (2008) present a broad overview of spatial interpolation methods in environmental science and their application in numerous studies. Babak and Deutsch (2009) stated that IDW interpolation and its modifications are the most frequently applied deterministic methods (without the authors citing any evidence for this). A number of publications compare IDW and Kriging as methods for the spatial interpolation of hydrochemical groundwater characteristics using various water quality parameters and scales (Elumalai et al. 2017; Gong et al. 2014; Mirzaei and Sakizadeh 2016; Mueller et al. 2004; Rostami et al. 2019; Zimmerman et al. 1999). For sample datasets with semi-variograms that did not indicate the presence of spatialautocorrelation, Mueller et al. (2004) conclude that IDW is a better choice than Ordinary Kriging. When performing an IDW procedure, the methodological problems are regularly discussed. Above all, it is not possible to derive measures of uncertainty from deterministic methods in addition to the estimates (Ohmer et al. 2017). When a deterministic criterion is used, the measures of optimality are chosen arbitrarily (Borga and Vizzaccaro 1997). IDW is very sensitive to the amount of data used in interpolation and to the exponent value (Kravchenko et al. 1999). Only very few studies deal with the interpolation of nitrate concentrations in groundwater using IDW. For a 4545 km^2^ district in India, Kriging outperformed IDW and other techniques to interpolate the spatio-seasonal variation of nitrate in the aquifers (Mukherjee and Singh 2021). The optimal IDW exponent values range between 1.30 and 1.54 for the interpolation of nitrate concentration in 41 groundwater sites in Greece (Charizopoulos et al. 2018). Of the six methods investigated, IDW shows the greatest mean absolute error in the interpolation of a vertical transect of nitrate concentration (Bronowicka-Mielniczuk et al. 2019). For the arsenic concentration in groundwater in Texas (USA), the correlation coefficient between the measured and estimated values with IDW was higher than with Kriging interpolation (Gong et al. 2014). The results of the interpolation of soil fertility data were better with determination of the IDW exponent based on an independent dataset instead of estimating IDW exponents by means of the minimization of cross-validated errors (Mueller et al. 2005). The large influence of data and sampling characteristics on the interpolation accuracy of IDW was highlighted by Zimmerman et al. (1999). The authors stated that the effect of certain data characteristics (such as the level of noise or the strength of spatial correlation) on interpolation accuracy can only be systematically evaluated with synthetic data.

Although this question is highly relevant for farmers in Germany, the uncertainty of IDW interpolation for the delimitation of nitrate-contaminated areas has not yet been examined. Our study quantifies the accuracy of IDW interpolations of nitrate concentration in groundwater by three approaches: (i) spatial correlation of nitrate concentration for 5790 groundwater monitoring sites, (ii) leave-one-out cross-validation of nitrate concentration for the 5790 sites, and (iii) Monte Carlo IDW interpolations of groundwater nitrate concentration based on a Random Forest modelled groundwater nitrate map as reference. The approaches are applied on a dataset for the federal state of Bavaria. The analysis focusses on the mean absolute error as the best measure to quantify the deviation between reference and interpolated nitrate concentrations. Furthermore, the performance of the IDW interpolation is evaluated with regard to the share of nitrate-contaminated areas within the region, i.e. the agricultural land where nitrogen fertilization must be reduced by 20% according to the Fertilizer Ordinance.

## Materials and methods

### Study area

**Fig. 1:**
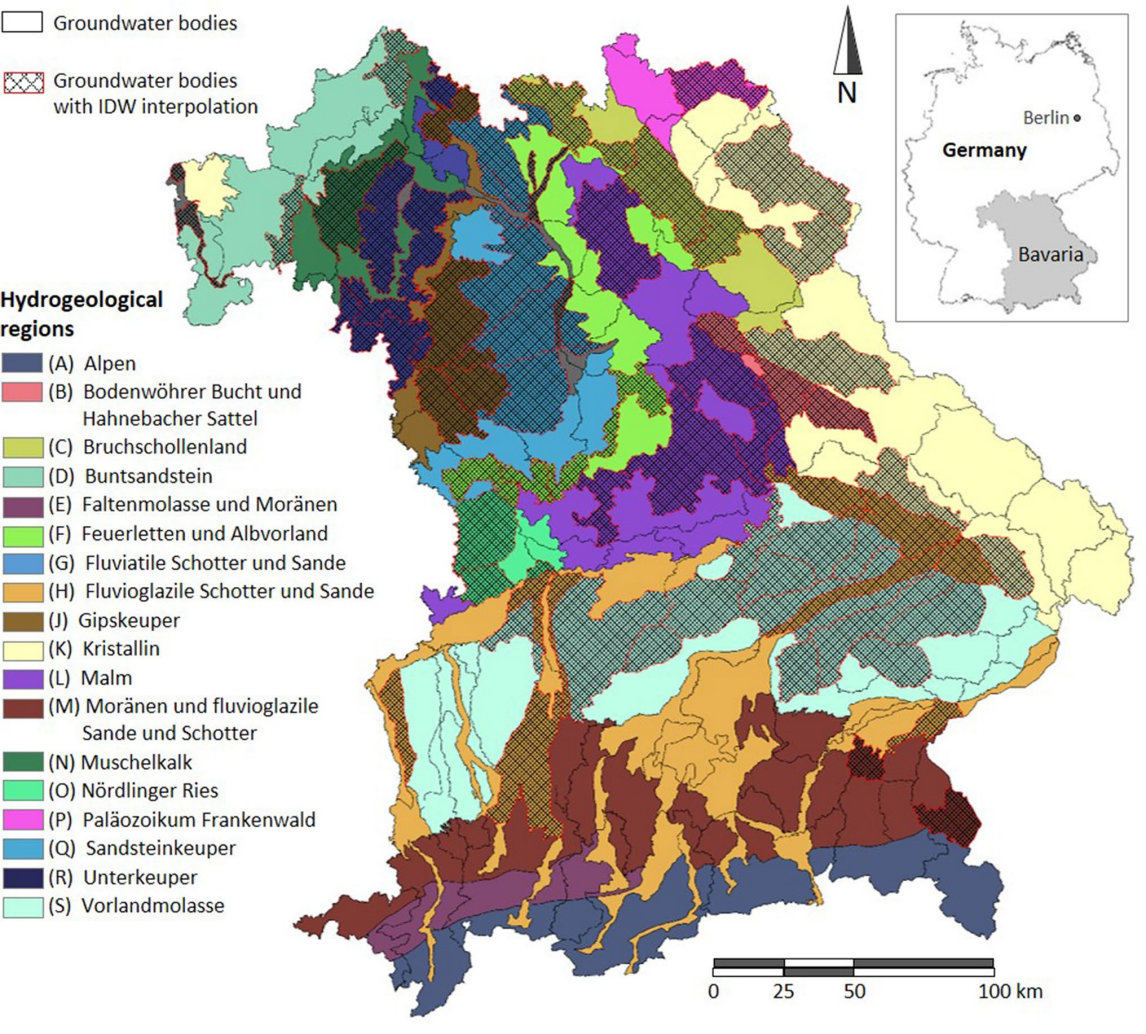
Hydrogeological regions (Hydrogeological map 1:500,000 Bavaria, LfU 2009) and groundwater bodies (LfU 2021) in Bavaria. © Bayerisches Landesamt für Umwelt

Bavaria is Germany’s largest federal state with an area of 70,542 km^2^. The Hydrogeological map 1:500,000 Bavaria (LfU 2009) shows 18 hydrogeological regions (Fig. 1; Supplement Table S1). In order to implement the WFD, 260 groundwater bodies are designated (LfU 2021), of which 77 are not of ‘good chemical status’ as per the WFD (hereafter referred to as ‘GWBs of concern’), which means they exhibit a nitrate concentration above 50 mg NO_3_/l or a concentration above 37.5 mg NO_3_/l in combination with a rising trend (Table S2).

We use a dataset from 7735 groundwater monitoring sites (measuring network and additional measuring sites) of the Bavarian State Office for the Environment. All sites are analysed at least one time per year. According to the AVV GeA (2020), the mean of the four annual maxima of nitrate concentrations for the period 2016 to 2019 is calculated by the Bavarian State Office and is used as the WFD evaluation nitrate concentration of each measuring site. For some sites, the period from 2011 to 2019 is analysed, and in individual cases, data from 2020 are also accounted. The dataset contains local clusters of measurement sites with identical nitrate concentrations. These are in most cases well galleries where all individual wells are listed separately in the monitoring database. The default parametrization of an IDW interpolation utilizes a given number of sampling points (usually 12 or less), so that there is a strong bull’s eye effect if the majority or all sampling points are located within close proximity of each other and share identical nitrate values (Ohmer et al. 2017). To avoid this effect, all measuring sites with an identical nitrate concentration that are located within the same 1 km × 1 km grid (see below) are combined to a single measuring site, which reduced the dataset to 5790 sites (Fig. S1 displays a map of the sites). A maximum of 32 sites within the same grid are combined to one single site.

For the spatial analysis, GWBs and hydrogeological regions are divided into 70,634 1 km × 1 km grids from the German GeoGrid map (BKG 2017) and measuring sites are assigned to their respective grids. Germany’s land cover model (LBM-DE2018, BKG 2019) is analysed with regard to the percentage of agricultural area (LBM-DE2018 code N211) of each grid. According to the LBM-DE2018, agricultural land covers 49.3% of the land area of Bavaria, whereas the Agricultural Census states that the share of agricultural land only amounts to 44.3% land area. The higher value is mainly due to the methodology of the LBM-DE2018, which sets a minimal mapping unit of 1 hectare. This results in the systematic underrepresentation of land use classes with a small fraction of land area and the overrepresentation of classes with high shares of land area (primarily agricultural land).

### Inverse distance weighting (IDW)

The IDW method is based on the assumption that the value of a groundwater quality parameter at observation points closer to the point of prediction is more similar to it than more distant points (Li and Heap 2008). IDW interpolation estimates the unknown value of $\widehat{Z}$ at the point *x*_*0*_ using the values of a given number of observation points *x*_*i*_ (or by a given radius of search neighbourhood), weighted by an inverse function of the distance between the unknown point and the observation

1 $$\widehat{Z}\left({x}_{0}\right)=\sum\nolimits_{i=1}^{n}{\lambda }_{i}Z({x}_{i})$$

where *λ*_*i*_ represents the weight function assigned to each observation point *x*_*i*_ and *Z*(*x*_*i*_) is the measured value at *x*_*i*_. The weights are determined as

2 $${\lambda }_{i}=\frac{{d}_{i}^{-p}}{\sum\nolimits_{i=1}^{n}{d}_{i}^{-p}}$$

where *d*_*i*_ is the Euclidian distance between the predicted point *x*_0_ and the observation point *x*_*i*_, *n* is the total number of observation points used in the interpolation of *x*_0_, and the exponent *p* decides how the weight decreases as the distance increases (Xie et al. 2011).

### Spatial correlation and cross-validation of measurement sites

An IDW interpolation is based on the assumption that the closer the two points are to each other, the more similar the nitrate concentration at a point to be interpolated is to the concentration at a measuring site (Li and Heap 2008). This assumption is checked for the 5790 measuring sites by calculating the difference in nitrate concentration as a function of the distance between the measuring sites. Cross-validation is a common statistical method to assess the model accuracy if a dataset cannot be split into training and validation data. In a leave-one-out cross-validation, each observed point is sequentially omitted and its value is predicted by IDW interpolation using the remaining measured points (Ohmer et al. 2017). We perform the analysis firstly for the entire 5790 measurement sites of Bavaria as a whole and secondly for the sites of the hydrogeological regions separately (i.e. no interpolation across the boundaries of a hydrogeological region) with IDW parameters set to *n* = 8 and *p* = 2. The difference between the measured and the interpolated nitrate concentration is calculated for each site. IDW interpolation is carried out with the software R vers. 4.0.2 and the package ‘gstat’ (vers. 2.0.6). The statistical metrics mean, median, minimum (MIN), maximum (MAX), standard deviation (SD), 5^th^–95^th^ interpercentile range (Q90), mean absolute error (MAE) and root mean square error (RMSE) of the differences are calculated according to Ohmer et al. (2017); furthermore, the area above the threshold of 50 mg NO_3_/l (AaT) is determined.

### Monte Carlo IDW interpolation of groundwater nitrate concentration

**Fig. 2:**
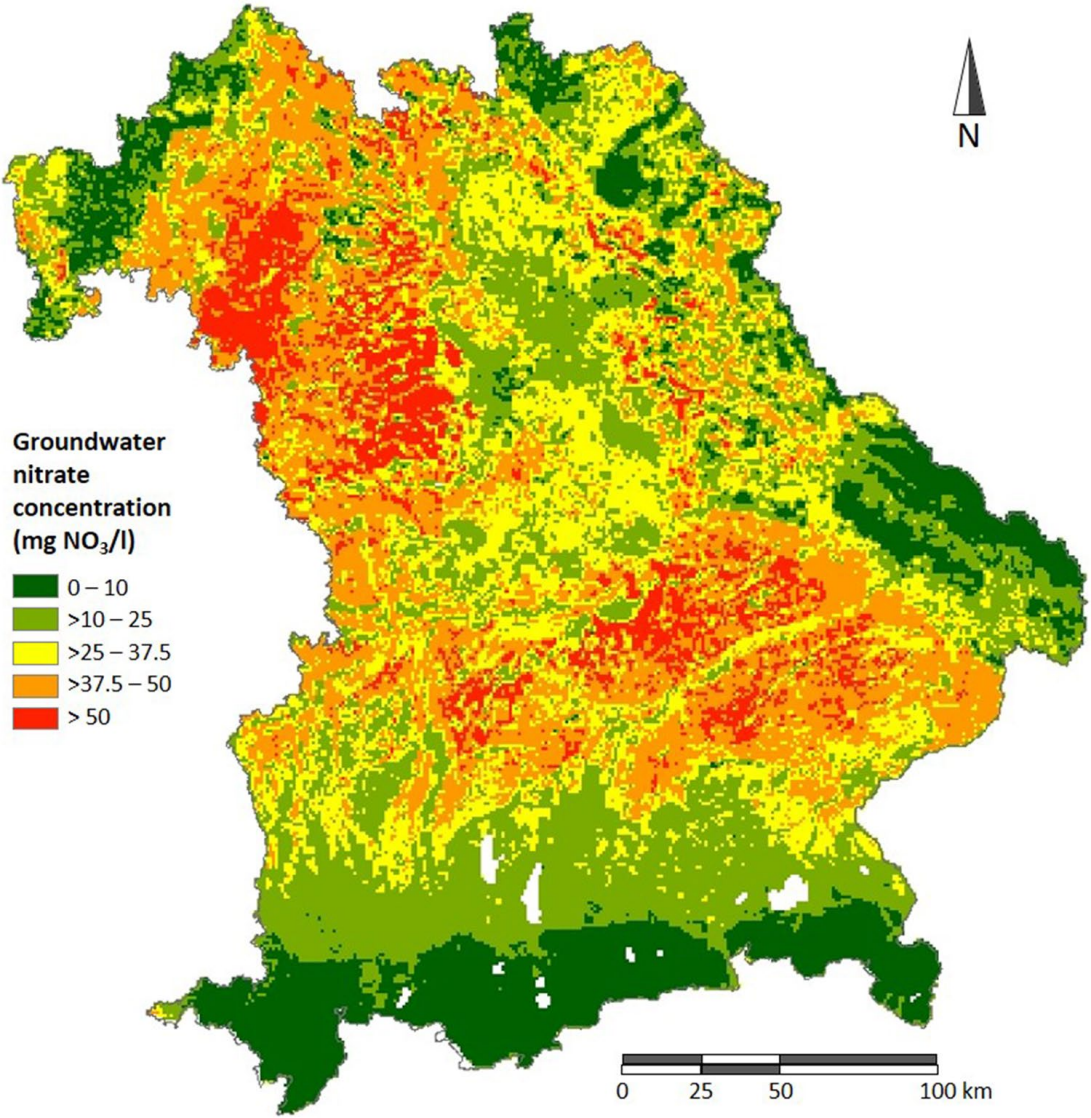
Random Forest model (1 km × 1 km) of groundwater nitrate concentration in Bavaria (Knoll et al. 2020). © GeoBasis-DE/BKG (2017) (Data license Germany www.govdata.de/dl-de/by-2-0)

The sensitivity and the accuracy of an IDW interpolation due to the variation of IDW parameters and the position of sampling points are analysed by a Monte Carlo (MC) IDW interpolation. Therefore, a map of groundwater nitrate concentrations as reference spatial distribution, which serves as basic population of the ‘true’ concentration values, is required. In the absence of any other data, we used a nationwide estimation of groundwater nitrate concentrations for Germany (1 km × 1 km grid) through Random Forest (RF) classification by Knoll et al. (2020). The predictive quality of this RF model (Fig. 2) achieves an *R*^2^ of 0.50; however, for its use as basic population of nitrate concentrations in Bavaria, the quality measure does not matter. From this RF map, a given number of grids is drawn randomly and their nitrate concentrations serve as sampling points for the IDW interpolation of the remaining grids, with the Euclidean distance calculated between the centres of the grid cells. This procedure is repeated 100 times and results in 100 interpolated nitrate concentration values for each grid, derived from 100 different spatial distributions of sampling points.

Firstly, the uncertainty of an IDW interpolation for the entire area of Bavaria is analysed with respect to three IDW parameters: the number *m* of total observation points (sampling point density), the number of sampling points *n* used for the individual grid interpolation, and the IDW exponent *p*; Table 1 gives the chosen parameter values. The three values of total observation points *m* correspond to sampling densities of one sampling point per 50, 20 and 10 km^2^, respectively related to the 70,634 grids of Bavaria. The 100 IDW interpolated maps are calculated for 70,634 minus *m* grids, without consideration of the affiliation of sampling points to hydrogeological regions. This approach was used to evaluate statistical metrics for the 3 × 4 × 4 = 48 parameter combinations.

**Table 1:** Parameter values of inverse distance weighting interpolation of groundwater nitrate concentration for Bavaria

Parameter		Values
*m*	Number of total observation points (out of 70,634 grids)	1412; 3531; 7063
*n*	Number of observation points used for weighting (Eq. 1)	4; 8; 12; 16
*p*	Exponent of the inverse distance weighting (Eq. 2)	1; 1.5; 2; 4

Secondly, 100 Monte Carlo IDW interpolations with randomly distributed sampling points are performed separately for 77 GWBs of concern. This approach is used to analyse the variability of the accuracy of an IDW interpolation for a larger number of small spatial units which differ in their hydrogeological and natural characteristics. The number of sampling points is not selected for all GWBs as a fixed proportion of the number of grids, but corresponds to the actual number of measuring sites in the GWBs (Table S2). IDW parameters are set as *n* = 8 and *p* = 2, and the maximum radius *maxdist* for sampling points used around the point of interpolation is confined to 10 km. Furthermore, only sampling points within the same hydrogeological region are considered, and no interpolation is performed across the boundaries of hydrogeological regions.

## Results

### Spatial correlation of measurement sites

**Fig. 3:**
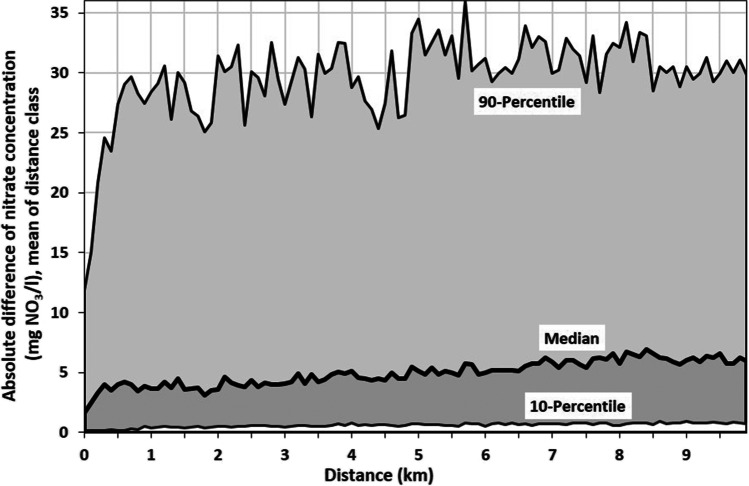
Median, 10^th^ percentile and 90^th^ percentile of the absolute differences of the nitrate concentrations of the 5790 monitoring sites in Bavaria as function of the distance of the monitoring sites, averaged for 100 m distance classes

A total of 103,531 concentration differences up to a distance of 10 km between two sites are analysed. The median, 10^th^ and 90^th^ percentile of the absolute nitrate concentration differences are calculated for 100 distance classes of 100 m each; the number of absolute differences in the classes ranges from 518 to 1726. The overall median of the absolute differences amounts to 4.9 mg NO_3_/l, only a weak dependency between nitrate concentrations and distances can be seen up to the distance class 0.3–0.4 km (Fig. 3; Fig. S2 presents a section enlargement for the distance up to 1.0 km).

### Cross-validation of nitrate concentration for measurement sites

**Fig. 4:**
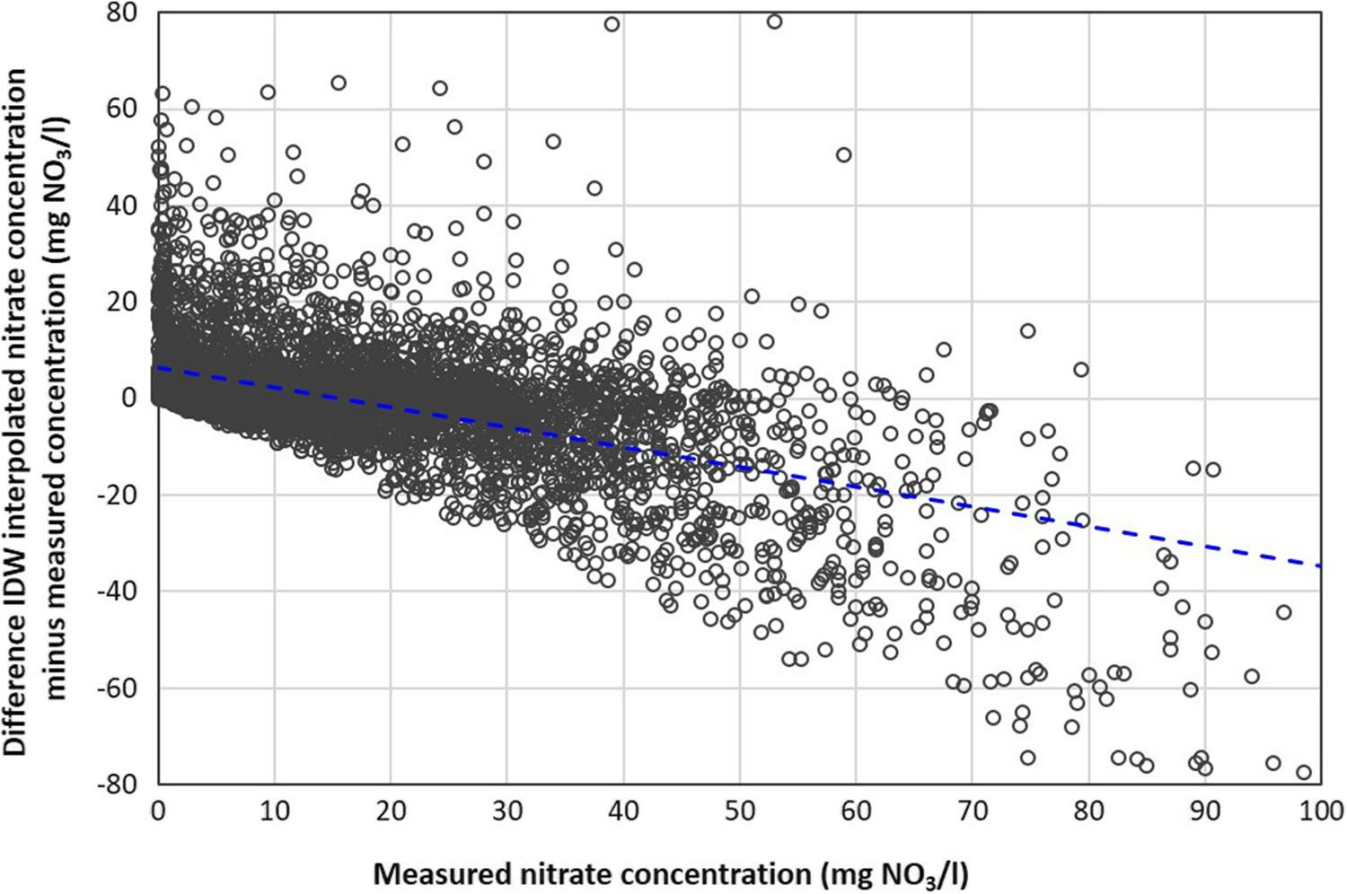
Difference between measured and inverse distance weighted (IDW) interpolated nitrate concentration for the 5790 measurement sites in Bavaria as function of measured concentration. IDW parameters *n* = 8, *p* = 2 and *maxdist* = 10; 32 data points* X* > 100, *Y* <  − 80 and *Y* > 80 not displayed

The overall mean nitrate concentration of the 5790 measurement sites is 16.6 mg NO_3_/l (Table 2). The average MAE between the measured and the IDW cross-validated nitrate concentrations amounts to 7.0 mg NO_3_/l for entire Bavaria; with regard to the mean concentration this corresponds to a relative MAE of nearly 42%. The correlation coefficient between measured and interpolated concentrations is 0.703. The distance to the eight neighbouring sites used in the IDW interpolation ranges on average between 918 m for the closest site and 4433 m for the furthest site (sites with a distance of more than 10 km are not included in the interpolation). The difference between measured and interpolated nitrate concentrations of the 5790 measurement sites shows a distinct dependence on the level of the measured concentration (Fig. 4). In the concentration range above approx. 17 mg NO_3_/l, the IDW interpolation systematically underestimates the true concentration. For the 277 sites with a measured nitrate concentration above 50 mg NO_3_/l, the cross-validated concentration is on average 28.6 mg NO_3_/l lower than the measured concentration, which corresponds to a mean relative error of 39%.

**Table 2:** Statistical measures of nitrate concentration of 5790 groundwater measurement sites and inverse distance weighted (IDW) interpolated concentrations for all sites in Bavaria (interpolated across the borders of the hydrogeological regions) and interpolated for the sites of the hydrogeological regions separately

Statistical measures	Measurement sites	IDW interpolation	
		All sites	HGR separately
Mean (mg NO_3_/l)	16.62	16.22	16.34
Minimum (mg NO_3_/l)	0.00	0.03	0.03
Maximum (mg NO_3_/l)	395.0	269.7	269.7
Standard deviation (mg NO_3_/l)	19.30	15.98	16.40
Mean absolute error (mg NO_3_/l)		7.03	7.11
Correlation coefficient		0.710	0.707
Number of sites with concentration above 50 mg NO_3_/l	277	154	171
-Percentage of true number		55.6%	61.7%

The tendency of the IDW method to narrow the distribution of nitrate concentrations is particularly evident with regard to the number of monitoring sites with concentrations above 50 mg NO_3_/l. While 277 monitoring sites are above 50 mg NO_3_/l, the IDW cross-validation reduced this number to 154 monitoring sites (55.6%) with interpolation for Bavaria as a whole, or to 171 monitoring sites (61.7%) with interpolation separately for the hydrogeological regions. The differences between measured and IDW interpolated nitrate concentrations show no correlation to the respective sum of weights Σ λ_i_ (Eq. 2), which means that an IDW interpolation based on sites close to the interpolated point does not fit better than an interpolation using more distant sites (within the search radius of 10 km).

The analyses of the cross-validation separately for the 18 hydrogeological regions illustrate the wide range of uncertainty of the IDW interpolation in different regions (Table S2). In this case, the MAE spans from 1.2 mg NO_3_/l in region A to 21.7 mg NO_3_/l in region O; the correlation coefficient ranges from 0.048 in region B to about 0.78 in regions G and H. Obviously, mainly hydrogeological and other factors determine the predictive quality of an IDW interpolation, but not the distance between the interpolation sites.

### Monte Carlo IDW interpolation of groundwater nitrate concentration in Bavaria

Table 3 shows the statistical measures of the IDW interpolation of the nitrate concentration in the 70,634 grids for the averaged levels of the parameters number of total observation points (*m*), number of observation points used for weighting (*n*), and IDW exponent (*p*), where the measure for each parameter combination represents the mean of 100 MC interpolations with randomly distributed sampling points (ref. to Table S3 for results of individual 48 parameter combinations). The mean of all parameter levels is nearly identical with the overall mean. A comparison of the minimum and maximum of the IDW interpolation with the population demonstrates a smoothing effect of IDW on the extremes at both ends of the spectrum. This effect increases with increasing number of observation points used for weighting and with decreasing IDW exponent. The standard deviation and the 5^th^–95^th^ interpercentile range vary slightly between parameter levels. Correlation between IDW maps and the reference map improves slightly with increasing number of total observation points and the MAE is reduced from 6.6 mg NO_3_/l with 1412 sampling points to 5.4 mg NO_3_/l with five times the density of sampling points. The statistical measures MAX and AaT depend most on the gradation of a parameter, i.e. if a larger IDW exponent is chosen, the smoothing effect of the IDW interpolation and the deviation of MAX and AaT from the reference values are reduced.

**Table 3:** Statistical measures of IDW interpolations of groundwater nitrate concentration in Bavaria, averaged over parameter number of total observation points (*m*), number of observation points used for weighting (*n*), and IDW exponent (*p*)

Parameter		Mean	MIN^a^	MAX	SD	Q90	MAE	RMSE	COR	AaT
	Value	mg NO_3_/l							-	%
*m*	1412	28.51	3.2	68.5	13.52	45.82	6.60	9.09	0.814	4.28
	3531	28.50	3.1	69.3	13.80	46.12	5.93	8.29	0.847	4.54
	7063	28.50	3.1	69.8	13.96	46.34	5.40	7.63	0.872	4.50
*n*	4	28.50	3.0	71.6	14.19	46.75	6.01	8.50	0.839	5.23
	8	28.51	3.1	69.3	13.79	46.14	5.94	8.31	0.845	4.48
	12	28.51	3.2	68.3	13.60	45.84	5.96	8.27	0.847	4.14
	16	28.51	3.2	67.5	13.47	45.65	5.99	8.27	0.847	3.92
*p*	1	28.52	3.3	64.3	13.32	45.44	6.05	8.29	0.845	3.65
	1.5	28.50	3.2	67.2	13.51	45.72	5.93	8.20	0.849	3.98
	2	28.51	3.1	70.1	13.74	46.06	5.88	8.21	0.849	4.39
	4	28.51	3.0	75.1	14.47	47.15	6.05	8.24	0.835	5.75
Reference population		28.49	2.7	81.1	15.53	48.77				8.29

^a^MIN: minimum; MAX: maximum; SD: standard deviation; Q90: 5^th^–95^th^ interpercentile range; MAE: mean absolute error; RMSE: root mean square error; COR: Pearson correlation coefficient; AaT: percentage area above threshold 50 mg NO_3_/l. Mean, SD, Q90, MAE: averaged over 16 (for *m*) or 12 (for *n*, *p*) parameter combinations × 100 interpolations × (70,634 − *m*) grids; MIN, MAX: minimum, maximum of 16 (for *m*) or 12 (for *n*, *p*) parameter combinations × 100 interpolations × (70,634 − *m*) grids; RMSE, COR, AaT: averaged over 16 (for *m*) or 12 (for *n*, *p*) parameter combinations × 100 interpolations

**Fig. 5:**
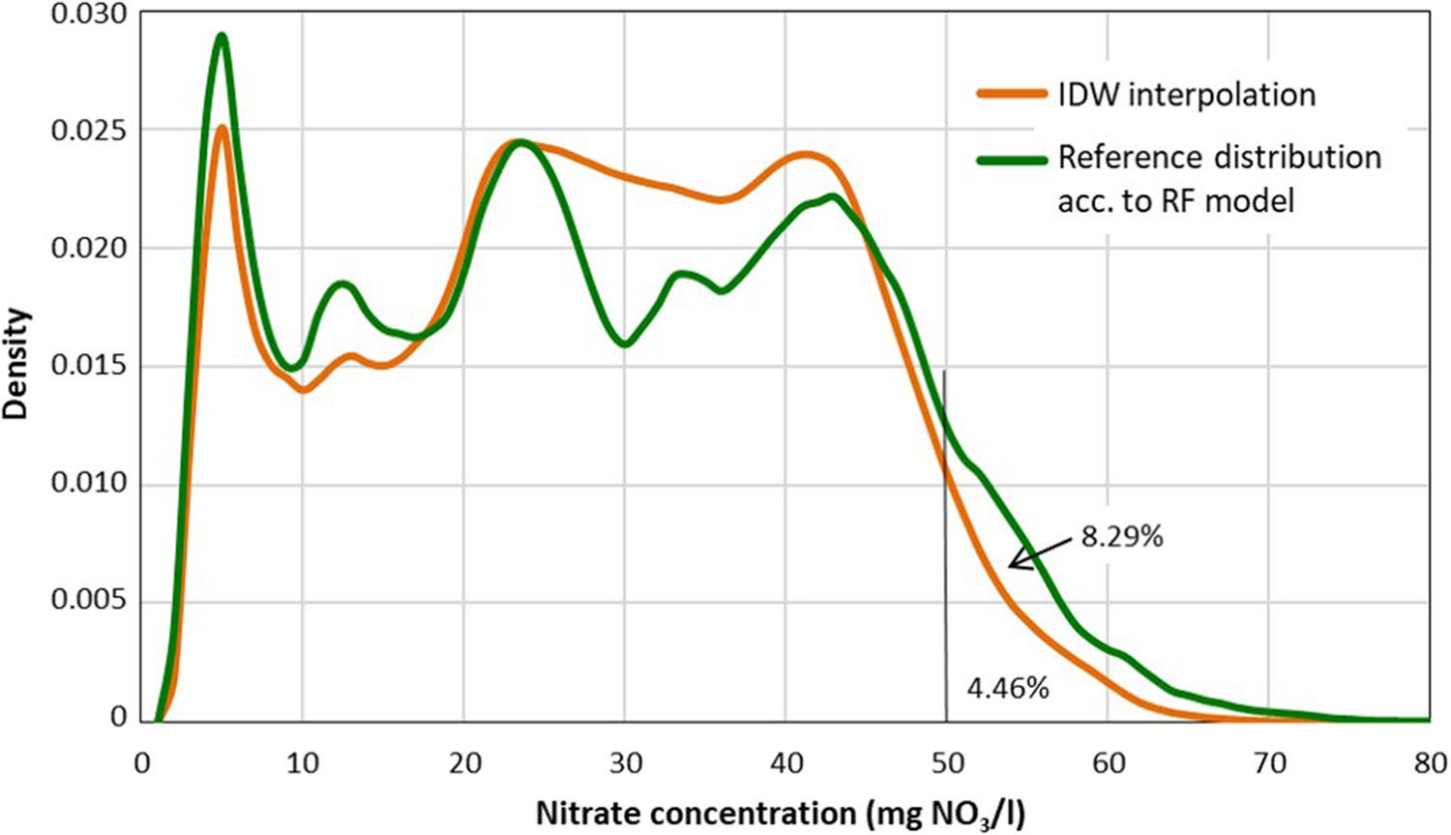
Frequency density of groundwater nitrate concentration in Bavaria (70,634 grids) from inverse distance weighting (IDW) interpolation (mean of 100 random allocations of 7063 sampling points) and from Random Forest (RF) model (Knoll et al. 2020); percentage values indicate the area above threshold of 50 mg NO_3_/l. IDW parameters *n* = 8, *p* = 2

The result of the IDW parameter combination *m* = 7063, *n* = 8 and *p* = 2 has the lowest MAE (ref. Table S3) and is analysed in more depth. Figure 5 gives the frequency density distributions of the reference concentration by the RF model and the IDW interpolation (as mean of 100 MC interpolations). Obviously, the IDW interpolation smooths the density distribution and reduces the variance of the basic population. The reference value of 8.3% of the area above 50 mg NO_3_/l in groundwater is more than 50% higher than the value of 4.46% by the IDW interpolation. The distribution of differences between IDW interpolated and reference nitrate concentrations as a function of the concentration for the IDW groundwater nitrate map of Bavaria (Fig. S3) shows a systematic mismatch analogous to the distribution of differences from cross-validation for the measurement sites (Fig. 4). The reference nitrate concentrations above the mean of approx. 28 mg NO_3_/l are systematically underestimated by the IDW prediction. For the 5859 grids above the threshold of 50 mg NO_3_/l according to RF modelling, IDW predictions of the concentration are on average 13.6 mg NO_3_/l too low.

The range (MIN–MAX) of the proportion of grids > 50 mg NO_3_/l from 100 MC IDW interpolations is around 1 percentage point (Table 4). As a result of the different positions of randomly selected sampling points, the groundwater area > 50 mg NO_3_/l estimated according to IDW interpolation can thus vary between nearly 4.0 and 5.0%. For the agricultural area (according to LBM-DE2018) in the grids > 50 mg NO_3_/l, the difference between the mean of IDW interpolations of approx. 6.3% and the value of the reference distribution of approx. 12.8% is even more pronounced, the relative underestimation for the agricultural area is approx. 51%.

**Table 4:** Mean, minimum and maximum of the number of grids (1 km × 1 km) with groundwater nitrate concentration above 50 mg NO_3_/l from 100 inverse distance weighting (IDW) interpolations with 7063 randomly selected sampling points each and values for the reference map (Random Forest model) for Bavaria

Area analysis	100 IDW interpolations^a^			Reference concentration map (RF model)
	Minimum	Mean	Maximum	
Number of grids > 50 mg NO_3_/l (= km^2^)	2801	3149	3521	5859
Share of state area Bavaria	3.97%	4.46%	4.98%	8.29%
Agricultural land area (km^2^) in grids > 50 mg NO_3_/l	1964	2195	2428	4467
Share of total agricultural area in Bavaria	5.64%	6.30%	6.97%	12.82%

^a^IDW parameters *m* = 7063, *n* = 8 and *p* = 2

The proportion of correctly interpolated areas alone is not exhaustive for assessing the quality of an interpolation procedure; rather, the matching localization is decisive. Therefore, the number of matching grids is determined which show a nitrate concentration above 50 mg NO_3_/l both in the reference distribution of the RF model and after the IDW interpolation, and the agricultural area (according to LBM-DE2018) within the matching grids (Table 5). In relation to the number of grids > 50 mg NO_3_/l according to IDW, this is around 67% each in the mean, minimum and maximum of the 100 interpolations. This means that the IDW interpolation not only underestimates the proportion of grids > 50 mg NO_3_/l, but also that only about 67% of these interpolated grids > 50 mg NO_3_/l are congruent with the reference population of the RF model; for the remaining 33% of the IDW grids > 50 mg NO_3_/l, the spatial allocation is incorrect. For the agricultural area in the matching grids, the result is similar, with about 74% correct and 26% incorrect localizations.

**Table 5:** Number of matching grids and agricultural areas in Bavaria (70,634 grids) with a nitrate concentration above 50 mg NO_3_/l after IDW interpolation (mean, minimum and maximum after 100 interpolations^a^) and in the reference distribution (Random Forest model)

Area analysis	100 IDW interpolations^a^		
	Minimum	Mean	Maximum
Number of grids > 50 mg NO_3_/l matching between IDW interpolation and reference concentration map in Bavaria (= km^2^)	1897	2115	2326
Agricultural land area (km^2^) in matching grids > 50 mg NO_3_/l	1456	1623	1780

^a^IDW parameters *m* = 7063, *n* = 8 and *p* = 2

The MC IDW spatial interpolation is also carried out separately for the 77 GWBs of concern, the number of randomly selected sampling points corresponds to the specific number of measurement sites (Table S2). This approach estimates the variability of an IDW accuracy at the level of the GWB. The mean MAE of the 77 GWB amounts to 7.0 mg NO_3_/l with the minimum value of a GWB at 3.7 mg NO_3_/l and the maximum MAE at 12.7 mg NO_3_/l.

## Discussion

The study focuses on how well IDW functions as an interpolation method to determine nitrate vulnerable zones under the EU Nitrates Directive. To the best of our knowledge, the usefulness of IDW for this purpose has not yet been examined. As an example, we analyse the accuracy of the IDW method for the federal state of Bavaria based on three approaches. In the first approach, we examine the absolute differences of nitrate concentrations between the 5790 measurement sites in Bavaria as a function of the distance, and find no spatial correlation of nitrate concentration beyond a range of about 400 m. Consequently, the use of IDW as an interpolation method is not justified from a statistical point of view for the designation of nitrate contaminated regions.

In the second approach, the accuracy of the IDW interpolation is examined by cross-validating the 5790 measurement sites. The essential evaluation parameter here is the mean absolute error, which quantifies the divergence (in mg NO_3_/l) between the actual and the interpolated nitrate concentrations, i.e. it characterizes the operational forecast quality of the IDW method. The MAE between the measured and the cross-validated interpolated nitrate concentrations amounts to 7.0 mg NO_3_/l on average for the 5790 monitoring sites, which corresponds to a relative MAE of about 42% with respect to the mean concentration of 16.6 mg NO_3_/l. Of the few studies on the interpolation of the nitrate concentration in groundwater in the literature, only Bronowicka-Mielniczuk et al. (2019) list a MAE that is significantly higher than in our application, at 18.9 mg NO_3_/l. IDW cannot predict values above or below the maximum and minimum measured values (Ohmer et al. 2017). The smoothing effect of an IDW interpolation causes a systematic underestimation of nitrate concentrations in the range of higher concentrations; the strength of the underestimation increases with increasing actual nitrate concentration. With regard to the number of monitoring sites with a concentration above the Nitrate Directive threshold of 50 mg NO_3_/l, the IDW cross-validation reduces the number of sites above the threshold from 277 actual sites to 154 sites in Bavaria (without consideration of hydrogeological regions). The MAE calculated separately for the measurement sites of the 18 hydrogeological regions illustrates the large spatial variability of the IDW accuracy. Obviously, the predictive capacity of an IDW interpolation also depends significantly on other factors such as hydrogeological features, land use and climate within an area of interest. However, investigating the possible effects of these factors was beyond the scope of this study.

The third approach investigates how well a given spatial distribution can be reproduced by an IDW interpolation. For this purpose, we regard a Random Forest map modelled by Knoll et al. (2020) as reference groundwater nitrate concentration in Bavaria, and the deviation of IDW interpolations to the reference map is analysed. A similar procedure was used by Zimmerman et al. (1999) and Babak and Deutsch (2009). In contrast to these publications, we do not perform a single IDW interpolation for each varied factor, but 100 repetitions with randomly distributed sample points, which allows us to capture the spread of uncertainty measures. The IDW parameters number of total sampling points, number of sampling points used for weighting, and IDW exponent (in the ranges examined here) only have minor effects on the quality of the interpolation compared to the variability due to varying positions of the sampling sites. The MAE for the 70,634 grids of Bavaria is on average about 6 mg NO_3_/l, with respect to the overall mean concentration of 28.5 mg NO_3_/l of the reference map; this is an overall relative MAE of 21%. With regard to the statistical measures MIN, MAX, MAE and the underestimation of the AaT, better results tend to be achieved; the larger the number of total sampling points (sampling density), the smaller the number of observation points used for weighting, and the higher the weighting exponent. Overall, however, the variation of the IDW parameters has only a relatively small influence on the MAEs of the maps derived from the individual parameter combinations.

For a specific IDW parameter combination, the spatial uncertainty is also evaluated in comparison to the reference nitrate concentration map. On the one hand, with IDW the area with a nitrate concentration above 50 mg NO_3_/l in Bavaria is under-interpolated by about 46% and for the agricultural land in Bavaria this amounts to about 51%. In the context of the designation of nitrate vulnerable zones, this means a considerable reduction of the areas for which groundwater protection programmes must be introduced under the Nitrates Directive. On the other hand, for some of the designated areas, their location does not correspond to the ‘true’ location of the polluted groundwater areas (according to the RF modelled reference map). As a consequence, farms may be affected by restrictions on nitrogen fertilization that are not necessary according to the ‘true’ distribution of groundwater nitrate concentration.

## Conclusion

We conclude that inverse distance weighting is not suitable as a regionalization method for the designation of nitrate vulnerable zones due to the systematic underestimation of higher nitrate concentrations and the insufficient identification of areas with nitrate concentrations above 50 mg NO_3_/l in groundwater.

## Supplementary Information

Below is the link to the electronic supplementary material.

Supplementary file1 (PDF 747 KB)

## Data Availability

The initial data on WFD relevant nitrate concentration in Bavarian groundwater measurement sites are available on request from the Bavarian State Office for the Environment (Bayerisches Landesamt für Umwelt, Hans-Högn-Straße 12, 95,030 Hof, Germany). The dataset generated during the current study is available from the corresponding author on reasonable request.
