# Supplementary material for: Accuracy assessment of inverse distance weighting interpolation of groundwater nitrate concentrations in Bavaria (Germany)
Source: Environ Sci Pollut Res Int. 2022 Sep 3;30(4):9445–55. doi: 10.1007/s11356-022-22670-0 (PMC9898373; doi:10.1007/s11356-022-22670-0)
Supplement: Supplementary file 1 — Supplementary file1 (PDF 747 KB) [file 11356_2022_22670_MOESM1_ESM.pdf]

Accuracy assessment of Inverse Distance Weighting interpolation of groundwater nitrate concentration in Bavaria (Germany)

Paul L. Ohlert[1], Martin Bach[1], Lukas Breuer[1,2]

[1] Institute for Landscape Ecology and Resources Management (ILR), Research Centre for Biosystems, Land Use and Nutrition (iFZ), Justus Liebig University Giessen, Heinrich-Buff-Ring 26, 35392 Giessen, Germany

[2] Centre for International Development and Environmental Research (ZEU), Justus Liebig University Giessen, Senckenbergstrasse 3, 35390 Giessen, Germany

Supplementary Information

List of Tables

Table S1 Hydrogeological regions in Bavaria (Hydrogeological map 1:500 000 Bavaria, LfU 2009), number of groundwater bodies (GWB) and number of measurement sites (MS) per region, and mean nitrate concentration. Mean absolute error (MAE) and Pearson correlation coefficient (COR) are calculated by cross-validation of measured and Inverse Distance Weighting interpolated nitrate concentration for the sites of the hydrogeological region.

Table S2 Location in hydrogeological region (Hy_ID), area, number of measurement sites (MS), site density, mean ‘true’ nitrate concentration (acc. to Random Forest modelling), standard deviation (SD) of ‘true’ concentration, mean absolute error (MAE) between ‘true’ and Inverse Distance Weighting (IDW) interpolated nitrate concentration, share of total agricultural land area and of agricultural land area in grids above 50 mg NO_3/l according to ‘true’ concentration vs IDW interpolation for 77 groundwater bodies (GWB) of concern in Bavaria.

Table S3 Statistical measures of Inverse Distance Weighting (IDW) interpolation of groundwater nitrate concentration in Bavaria (70,634 grids 1 km x 1km) for individual combinations of the IDW parameters: number of total observation points (m), number of observation points used for weighting (n), and IDW exponent (p).

List of Figures

Fig. S1 Map of the aggregated 5790 groundwater measurement sites used for measuring nitrate concentrations and the boundaries of the hydrogeological regions in Bavaria. Colouring corresponds to the measured nitrate concentrations.

Fig. S2 Absolute differences between actual and IDW cross-validated nitrate concentrations of the 5790 monitoring sites in Bavaria as function of the distance of the monitoring sites; median, 10- and 90-percentile averaged for 100 m distance classes from 0-0.1 km to 0.9-1.0 km (section enlargement from Fig. 3 in the main publication).

Fig. S3 Difference between Inverse Distance Weighting interpolated groundwater nitrate con-centration and ‘true’ concentration from Random Forest (RF) model in Bavaria (70,634 grids 1 km x 1 km) as function of ‘true’ concentration.

Table S1 Hydrogeological regions in Bavaria (Hydrogeological map 1:500 000 Bavaria, LfU 2009), number of groundwater bodies (GWB) and number of measurement sites (MS) per region, and mean nitrate concentration. Mean absolute error (MAE) and Pearson correlation coefficient (COR) are calculated by cross-validation of measured and Inverse Distance Weighting interpolated nitrate concentration for the sites of the hydrogeological region.

**Hy_ID**	**Hydrogeological region**	**Area**	**No. GWB**	**No. MS**	**Cross validation results**		
					**Mean conc.**	**MAE**	**COR**
		km²			mg NO_3/l	mg NO_3/l	
A	Alpen	3933	9	356	3.8	1.2	0.669
B	Bodenwöhrer Bucht und Hahnbacher Sattel	744	5	67	11.0	12.1	0.048
C	Bruchschollenland	2531	7	232	16.6	10.3	0.525
D	Buntsandstein	3094	10	312	11.4	5.6	0.709
E	Faltenmolasse und Moränen	1246	7	104	5.8	1.7	0.714
F	Feuerletten und Albvorland	2967	14	181	13.3	6.5	0.449
G	Fluviatile Schotter und Sande	605	10	203	22.1	9.2	0.783
H	Fluviatile und fluvioglaziale Schotter und Sande	9152	41	920	18.3	4.6	0.778
J	Gipskeuper	2285	10	93	29.2	18.5	0.089
K	Kristallin	9765	22	1251	9.4	4.2	0.657
L	Malm	6355	17	262	22.8	6.7	0.652
M	Moränen und fluvioglaziale Schotter und Sande	6407	28	648	14.5	4.1	0.729
N	Muschelkalk	1787	8	217	23.9	7.2	0.728
O	Nördlinger Ries	1128	6	48	31.1	21.7	0.103
P	Paläozoikum des Frankenwaldes	1021	5	83	10.2	7.3	0.370
Q	Sandsteinkeuper	4432	14	418	32.6	17.4	0.708
R	Unterkeuper	2151	7	76	28.5	17.0	0.485
S	Vorlandmolasse	10952	33	334	27.9	15.5	0.451
**Total**		**70555**	**253**	**5790**	**16.3**	**7.1**	**0.707**

Table S2 Location in hydrogeological region (Hy_ID), area, number of measurement sites (MS), site density, mean 'true' nitrate concentration (acc. to Random Forest modelling), standard deviation (SD) of 'true' concentration, mean absolute error (MAE) between 'true' and Inverse Distance Weighting (IDW) interpolated nitrate concentration, share of total agricultural land area and of agricultural land area in grids above 50 mg NO_3/l according to 'true' concentration vs IDW interpolation for 77 groundwater bodies (GWB) of concern in Bavaria.

GWB code	Hy_ID[a]	Area	No. of MS	MS density	Mean conc.	SD	MAE	Share of agricult. land area		
								Total	>50 mg NO_3/l	
									true	IDW
		km²		km²/MS	mg NO_3/	mg NO_3/	mg NO_3/l	%	%	%
1_G009	H	216.7	9	24.1	33.7	8.02	6.19	68.45	0.0	0.0
1_G022	O	201.6	9	22.4	33.2	11.01	9.63	49.96	6.0	0.7
1_G024	H	162.8	19	8.6	36.3	6.54	5.15	72.70	1.2	0.1
1_G025	O	41.8	1	41.8	29.7	9.09	10.69	47.07	0.0	0.0
1_G026	S	206.4	3	68.8	45.0	6.96	6.76	71.47	19.5	13.9
1_G028	F	252.2	1	252.2	40.5	8.63	9.90	65.56	4.3	0.6
1_G031	O	541.3	24	22.6	40.2	9.02	6.23	74.27	5.7	0.7
1_G040	H	819.5	79	10.4	27.8	9.37	5.13	64.59	0.0	0.0
1_G044	H	160.1	10	16.0	39.4	7.76	5.84	72.45	2.5	0.3
1_G050	S	1142.1	21	54.4	42.8	11.22	10.12	63.45	29.0	17.2
1_G053	S	505.9	14	36.1	48.1	13.34	11.19	64.87	41.5	39.7
1_G054	S	266.4	7	38.1	49.0	13.81	12.25	64.25	53.0	44.1
1_G055	S	244.2	3	81.4	37.7	6.30	5.54	64.07	0.8	0.1
1_G057	F	152.1	7	21.7	42.8	6.59	5.51	75.82	11.1	2.6
1_G058	J	364.7	12	30.4	43.6	8.12	6.82	67.96	18.6	8.8
1_G059	L	277.3	9	30.8	32.2	5.68	4.13	70.30	0.0	0.0
1_G061	L	1061.3	38	27.9	31.1	5.26	4.62	60.00	0.1	0.0
1_G066	C	305.8	25	12.2	36.4	12.78	9.67	61.51	15.0	4.6
1_G068	K	457.9	34	13.5	30.5	12.62	7.55	54.60	0.0	0.0
1_G070	B	252.9	22	11.5	33.9	14.68	9.76	38.08	21.6	6.7
1_G072	K	752.8	32	23.5	30.1	10.57	8.68	54.64	1.2	0.0
1_G074	L	290.2	6	48.4	31.8	8.64	8.04	43.84	3.5	0.4
1_G075	B	167.8	6	28.0	36.7	12.58	11.19	52.41	17.9	5.9
1_G077	L	562.7	21	26.8	28.2	8.34	6.54	44.43	2.5	0.1
1_G079	B	272.6	30	9.1	26.6	13.12	8.73	33.22	5.5	0.1
1_G082	L	117.0	9	13.0	40.3	8.90	7.74	54.01	14.9	1.0
1_G083	H	306.0	13	23.5	41.3	8.28	6.17	67.52	5.1	0.4
1_G086	H	435.7	27	16.1	40.4	7.57	4.34	77.64	1.8	0.2
1_G087	K	395.4	24	16.5	24.4	8.16	5.12	53.50	0.0	0.0
1_G089	S	223.8	4	56.0	48.8	6.96	5.87	75.18	53.2	46.1
1_G090	H	31.4	1	31.4	43.6	8.62	9.30	76.87	13.3	14.5
1_G091	S	481.2	11	43.7	44.2	9.37	8.51	66.87	30.8	13.0
1_G092	S	339.2	25	13.6	50.5	6.45	4.78	68.16	56.1	54.2
1_G105	H	367.4	32	11.5	35.5	7.30	5.22	68.45	0.8	0.0
1_G106	S	276.6	9	30.7	45.3	7.40	6.43	61.06	23.9	14.9
1_G107	S	404.1	19	21.3	47.4	6.78	5.63	64.65	38.2	26.1
1_G119	H	243.6	7	34.8	39.9	6.55	4.15	79.07	0.8	0.1
1_G120	K	301.5	2	150.8	27.1	5.62	5.54	54.91	0.0	0.0

Table S2 (continued)

GWB code	Hy_ID	Area	No. of MS	MS density	Mean conc.	SD	MAE	Share of agricult. land area		
								Total	>50 mg NO_3/l	
									true	IDW
		km²		km²/MS	mg NO_3/	mg NO_3/	mg NO_3/l	%	%	%
1_G122	S	572.7	41	14.0	46.7	8.17	6.07	70.14	38.2	25.3
1_G145	M	153.5	9	17.1	30.6	5.02	3.80	72.46	0.0	0.0
1_G151	H	171.8	10	17.2	27.0	8.41	7.03	38.68	0.0	0.0
1_G162	M	293.3	20	14.7	16.3	5.75	3.65	54.12	0.0	0.0
1_G165	B	17.2	3	5.7	32.9	10.75	10.11	44.05	5.9	0.7
2_G004	F	424.2	14	30.3	32.4	10.52	7.67	53.81	1.6	0.9
2_G005	J	307.3	18	17.1	46.1	10.53	8.29	55.00	36.4	26.4
2_G007	Q	655.1	84	7.8	52.3	12.04	8.56	55.11	68.0	62.1
2_G016	G	63.0	51	1.2	30.2	8.38	5.18	50.47	1.7	0.0
2_G017	J	86.4	6	14.4	43.8	7.42	6.37	61.14	19.0	6.6
2_G018	Q	572.4	37	15.5	46.9	13.08	8.80	52.98	54.6	44.7
2_G019	Q	119.3	37	3.2	31.5	16.79	6.39	26.74	24.0	11.2
2_G022	L	789.2	32	24.7	25.7	5.72	3.78	49.89	0.0	0.0
2_G025	J	620.7	32	19.4	42.5	10.39	7.36	61.48	19.9	9.8
2_G027	Q	725.2	61	11.9	44.3	10.24	8.34	54.15	37.5	17.0
2_G028	R	51.3	1	51.3	51.1	5.37	6.42	85.92	53.1	52.7
2_G030	C	679.9	57	11.9	33.0	10.72	7.88	56.15	5.8	0.5
2_G035	C	401.8	39	10.3	34.6	13.01	8.90	52.76	9.6	0.3
2_G037	G	102.6	8	12.8	36.9	8.85	6.45	61.18	3.8	0.6
2_G039	Q	552.3	45	12.3	36.6	14.50	10.50	44.88	16.6	2.6
2_G044	Q	101.1	6	16.9	34.2	14.69	12.73	37.80	10.6	4.0
2_G045	Q	49.1	5	9.8	36.2	14.18	10.34	43.11	13.0	4.0
2_G046	R	558.2	36	15.5	49.5	9.21	5.51	74.61	48.5	46.2
2_G048	R	707.2	12	58.9	53.3	8.29	6.64	77.46	67.1	64.2
2_G055	N	591.8	62	9.5	39.0	9.38	5.65	55.65	4.6	0.6
2_G057	D	183.6	25	7.3	28.4	12.51	7.61	44.27	0.0	0.0
2_G062	G	114.2	32	3.6	34.0	8.49	4.98	43.84	0.9	0.0
2_G066	G	29.5	11	2.7	28.3	7.38	5.17	18.47	0.0	0.0
2_G067	D	250.6	25	10	30.5	10.75	6.97	52.78	0.0	0.0
2_G070	J	233.2	3	77.7	41.9	8.30	7.22	70.31	4.7	2.6
2_G073	R	116.7	12	9.7	43.9	8.86	6.26	73.90	20.7	6.8
2_G077	R	472.6	5	94.5	51.3	8.21	7.40	79.00	63.6	56.4
2_G079	C	116.3	9	12.9	35.9	10.92	8.92	56.29	6.8	0.9
2_G084	Q	36.6	18	2.0	29.4	14.73	4.66	17.65	19.4	5.1
5_G001	K	920.3	165	5.6	21.7	10.97	6.45	42.62	0.6	0.0
5_G007	P	395.3	43	9.2	29.4	9.43	6.08	57.42	0.0	0.0
Total / Mean		27013.9	1553	17.4	37.9	13.07	6.99	60.02	18.1	12.7

[a] Codes Hy_ID refer to Table S1.

Table S3 Statistical measures of Inverse Distance Weighting (IDW) interpolation of groundwater nitrate concentration in Bavaria (70,634 grids 1 km x 1km) for individual combinations of the IDW parameters number of total observation points (*m*), number of observation points used for weighting (*n*), and IDW exponent (*p*).

**Parameters**			**Mean**	**MIN**[a]	**MAX**	**SD**	**Q90**	**MAE**	**RMSE**	**COR**	**AaT**
m	***n***	***p***	mg NO_3/l	mg NO_3/l	mg NO_3/l	mg NO_3/l	mg NO_3/l	mg NO_3/l	mg NO_3/l		**%**
**1412**	**4**	**1**	28.54	3.16	67.27	13.70	46.06	6.61	9.12	0.812	4.75
		**1.5**	28.44	3.09	69.05	13.80	46.16	6.62	9.19	0.810	4.87
		**2**	28.51	3.04	71.20	14.02	46.51	6.63	9.26	0.808	5.29
		**4**	28.47	3.01	73.31	14.49	47.20	6.88	9.73	0.792	6.18
	**8**	**1**	28.50	3.30	63.36	13.03	45.03	6.55	8.88	0.821	3.40
		**1.5**	28.50	3.19	67.19	13.30	45.46	6.47	8.87	0.822	3.89
		**2**	28.50	3.10	70.47	13.55	45.82	6.49	8.97	0.818	4.40
		**4**	28.54	3.01	73.41	14.38	47.09	6.76	9.55	0.799	6.00
	**12**	**1**	28.48	3.47	61.49	12.71	44.65	6.61	8.85	0.822	2.83
		**1.5**	28.51	3.26	66.14	13.02	45.11	6.47	8.79	0.825	3.38
		**2**	28.53	3.12	70.13	13.35	45.52	6.44	8.86	0.822	4.01
		**4**	28.52	3.02	73.67	14.28	46.86	6.74	9.49	0.800	5.76
	**16**	**1**	28.57	3.60	60.19	12.50	44.54	6.66	8.87	0.821	2.56
		**1.5**	28.55	3.33	65.22	12.83	44.90	6.49	8.76	0.826	3.14
		**2**	28.49	3.16	69.44	13.17	45.27	6.46	8.82	0.823	3.63
		**4**	28.56	2.98	73.93	14.24	46.87	6.73	9.48	0.801	5.73
**3531**	**4**	**1**	28.51	3.09	68.36	13.94	46.40	5.95	8.35	0.845	5.05
		**1.5**	28.50	3.04	70.60	14.06	46.57	5.91	8.34	0.845	5.28
		**2**	28.50	3.02	72.30	14.20	46.76	5.91	8.39	0.844	5.54
		**4**	28.49	2.92	75.90	14.64	47.43	6.09	8.76	0.833	6.46
	**8**	**1**	28.52	3.21	64.31	13.41	45.55	5.95	8.19	0.850	4.00
		**1.5**	28.51	3.14	67.45	13.59	45.81	5.84	8.12	0.853	4.36
		**2**	28.52	3.07	70.00	13.82	46.18	5.80	8.14	0.853	4.77
		**4**	28.52	2.94	75.40	14.50	47.19	6.00	8.60	0.838	6.15
	**12**	**1**	28.51	3.29	62.73	13.17	45.15	6.02	8.20	0.849	3.57
		**1.5**	28.50	3.21	66.01	13.36	45.42	5.86	8.08	0.854	3.92
		**2**	28.51	3.10	69.66	13.63	45.88	5.78	8.06	0.855	4.42
		**4**	28.50	2.94	75.81	14.43	47.08	5.96	8.54	0.840	6.03
	**16**	**1**	28.51	3.36	61.37	12.96	44.79	6.09	8.25	0.847	3.24
		**1.5**	28.50	3.25	64.91	13.21	45.19	5.89	8.07	0.854	3.64
		**2**	28.49	3.13	68.85	13.48	45.58	5.80	8.04	0.855	4.13
		**4**	28.48	2.94	75.20	14.40	47.01	5.96	8.52	0.840	5.92
**7063**	**4**	**1**	28.52	3.05	70.31	14.11	46.66	5.42	7.69	0.870	5.33
		**1.5**	28.51	3.03	71.29	14.21	46.78	5.35	7.64	0.872	5.51
		**2**	28.52	2.99	72.78	14.33	46.97	5.33	7.64	0.872	5.75
		**4**	28.50	2.91	76.26	14.71	47.51	5.42	7.90	0.865	6.56
	**8**	**1**	28.51	3.15	65.98	13.63	45.83	5.49	7.64	0.871	4.29
		**1.5**	28.50	3.10	67.82	13.78	46.07	5.35	7.50	0.876	4.59
		**2**	28.51	3.05	70.16	13.95	46.35	5.28	7.47	0.877	4.95
		**4**	28.51	2.92	76.24	14.57	47.27	5.35	7.75	0.869	6.23

Parameters			Mean	MIN[a]	MAX	SD	Q90	MAE	RMSE	COR	AaT
m	*n*	*p*	mg NO_3/l	mg NO_3/l	mg NO_3/l	mg NO_3/l	mg NO_3/l	mg NO_3/l	mg NO_3/l		%
	**12**	**1**	28.51	3.21	63.50	13.40	45.46	5.59	7.70	0.869	3.89
		**1.5**	28.50	3.16	66.06	13.57	45.74	5.40	7.51	0.875	4.19
		**2**	28.51	3.09	68.63	13.78	46.07	5.28	7.42	0.878	4.64
		**4**	28.49	2.92	75.94	14.51	47.17	5.33	7.71	0.870	6.11
	**16**	**1**	28.52	3.26	62.32	13.25	45.23	5.67	7.77	0.866	3.67
		**1.5**	28.48	3.21	64.80	13.42	45.45	5.46	7.54	0.874	3.92
		**2**	28.50	3.13	68.05	13.66	45.83	5.30	7.42	0.879	4.39
		**4**	28.49	2.94	75.97	14.48	47.11	5.33	7.70	0.871	6.02
‚True‘ population			28.49	2.74	81.09	15.53	48.77				8.29

[a] MIN: minimum; MAX: maximum; SD: standard deviation; Q90: 5th-95th-interpercentile range; MAE: mean absolute error; RMSE: root mean square error; COR: Pearson correlation coefficient; AaT: area above threshold 50 mg NO_3/l. Mean, SD, Q90, MAE: averaged over 100 interpolations x (70,634 – *m*) grids; MIN, MAX: minimum, maximum of 100 interpolations x (70,634 – *m*) grids; RMSE, COR, AaT: averaged over 100 interpolations.

Fig. S1 Map of the aggregated 5790 groundwater measurement sites used for measuring nitrate concentrations and the boundaries of the hydrogeological regions in Bavaria. Colouring corresponds to the measured nitrate concentrations. © Bayerisches Landesamt für Umwelt

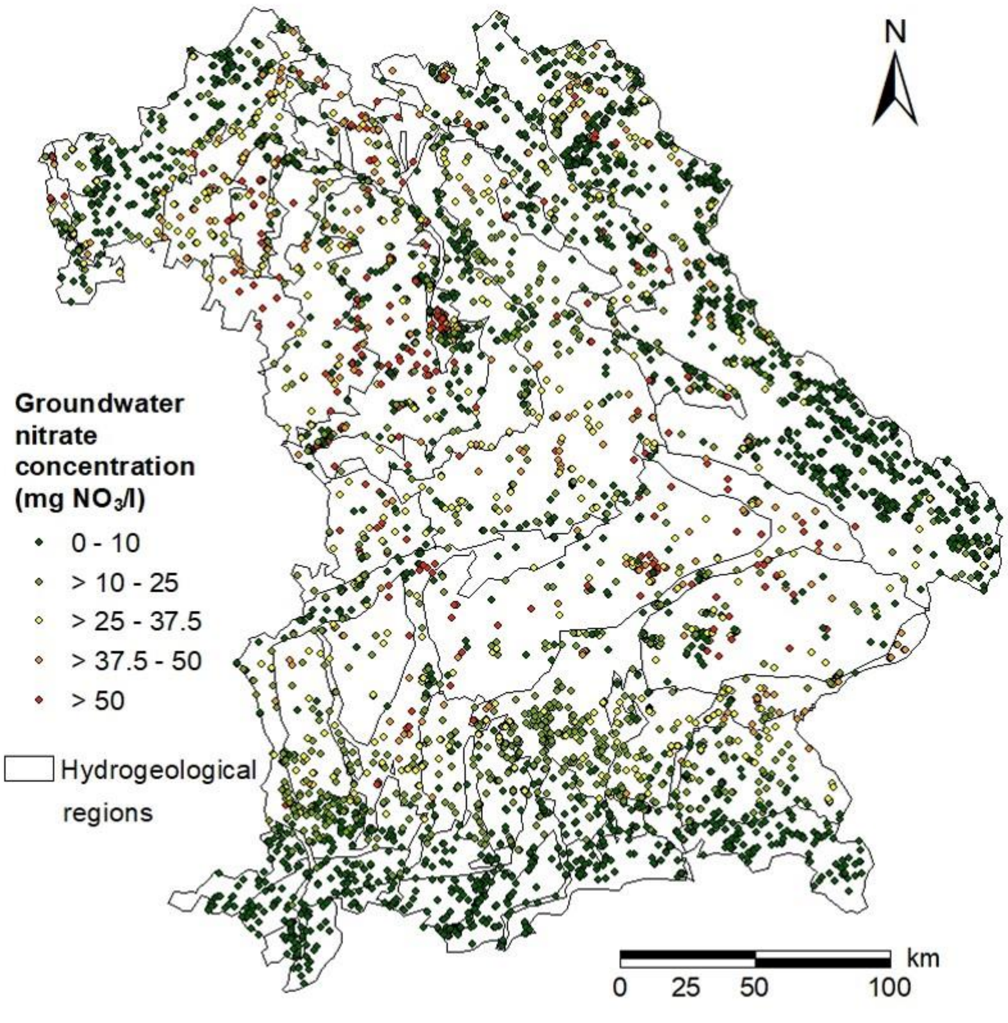

Fig. S2 Absolute differences between actual and IDW cross-validated nitrate concentrations of the 5790 monitoring sites in Bavaria as function of the distance of the monitoring sites; median, 10- and 90-percentile averaged for 100 m distance classes from 0-0.1 km to 0.9-1.0 km (section enlargement from Fig. 3 in the main publication).

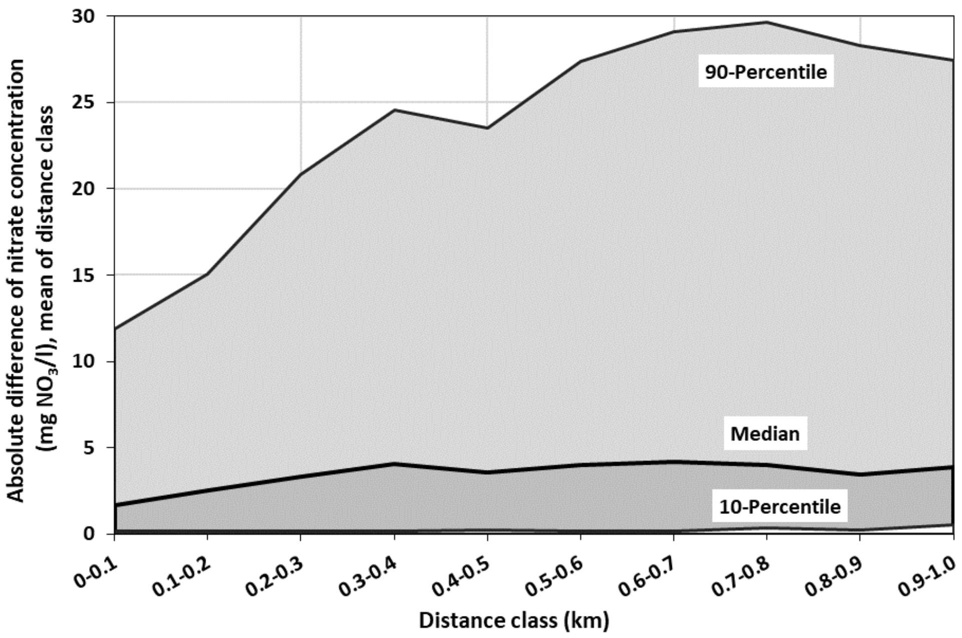

Fig. S3 Difference[a] between Inverse Distance Weighting (IDW) interpolated groundwater nitrate concentration and 'true' concentration from Random Forest (RF) model in Bavaria (70,634 grids 1 km x 1 km) as function of 'true' concentration.

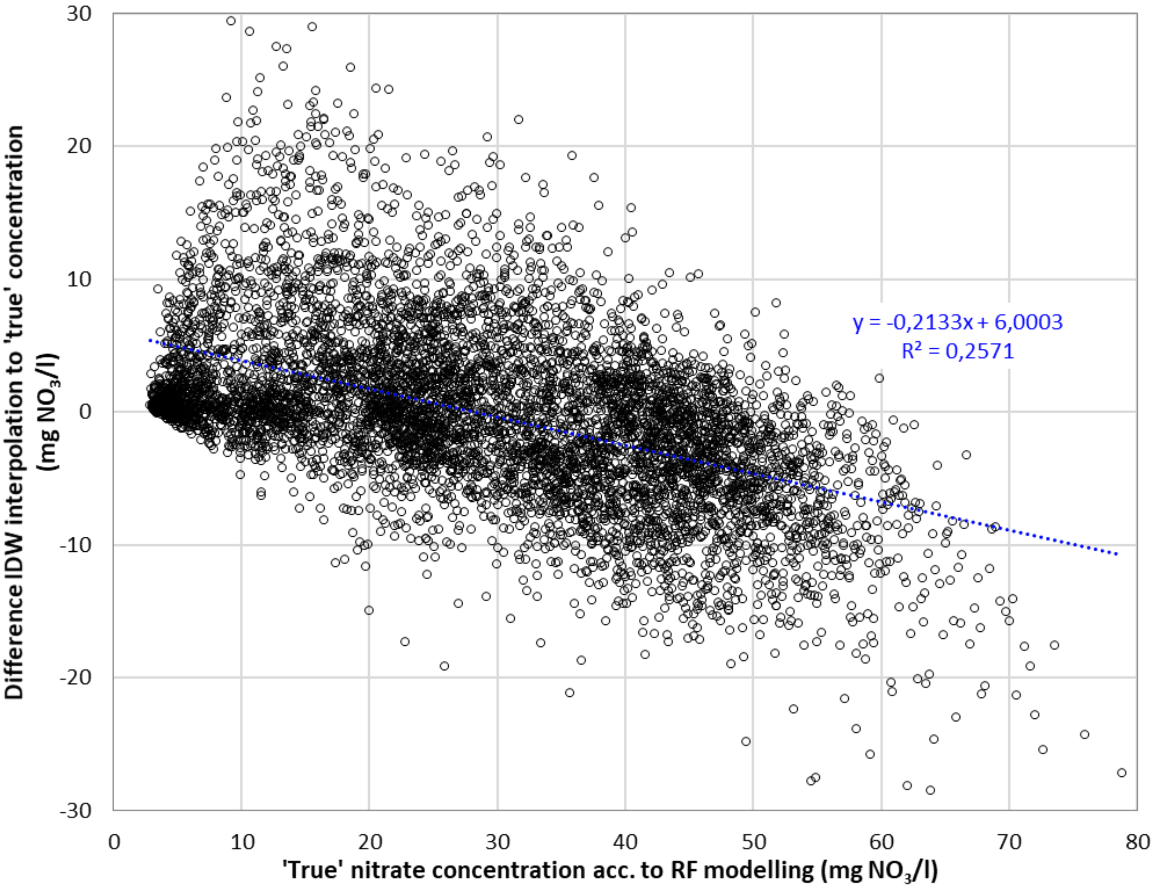

[a] Mean of 100 Monte Carlo IDW interpolations with parameter combination m = 7063, n = 8, and p = 2. The graphic only shows a random selection of 10% of the data points; data points Y <-30 and Y >30 not displayed.
